# Towards standardisation of contact and contactless electrical measurements of CVD graphene at the macro-, micro- and nano-scale

**DOI:** 10.1038/s41598-020-59851-1

**Published:** 2020-02-21

**Authors:** Christos Melios, Nathaniel Huang, Luca Callegaro, Alba Centeno, Alessandro Cultrera, Alvaro Cordon, Vishal Panchal, Israel Arnedo, Albert Redo-Sanchez, David Etayo, Montserrat Fernandez, Alex Lopez, Sergiy Rozhko, Oihana Txoperena, Amaia Zurutuza, Olga Kazakova

**Affiliations:** 1 National Physical Laboratory, Teddington, TW11 0LW United Kingdom; 2 Department of Electrical and Computer Engineering, Faculty of Engineering, University of Cyprus, 1687 Nicosia, Cyprus; 3 Istituto Nazionale di Ricerca Metrologica, Strada delle Cacce 91, 10135 Torino, Italy; 4 Graphenea SA, 20018 Donostia-San Sebastián, Spain; 5 Das-Nano, Poligono Industrial Talluntxe II, Calle M-10, 31192 Tajonar, Navarra Spain

**Keywords:** Electronic properties and devices, Imaging techniques, Characterization and analytical techniques, Materials science, Nanoscience and technology, Physics

## Abstract

Graphene has become the focus of extensive research efforts and it can now be produced in wafer-scale. For the development of next generation graphene-based electronic components, electrical characterization of graphene is imperative and requires the measurement of work function, sheet resistance, carrier concentration and mobility in both macro-, micro- and nano-scale. Moreover, commercial applications of graphene require fast and large-area mapping of electrical properties, rather than obtaining a single point value, which should be ideally achieved by a contactless measurement technique. We demonstrate a comprehensive methodology for measurements of the electrical properties of graphene that ranges from nano- to macro- scales, while balancing the acquisition time and maintaining the robust quality control and reproducibility between contact and contactless methods. The electrical characterisation is achieved by using a combination of techniques, including magneto-transport in the van der Pauw geometry, THz time-domain spectroscopy mapping and calibrated Kelvin probe force microscopy. The results exhibit excellent agreement between the different techniques. Moreover, we highlight the need for standardized electrical measurements in highly controlled environmental conditions and the application of appropriate weighting functions.

## Introduction

The unique properties of graphene^1^ and the increasing demand for large-scale production were the reason for the development of various industrial methods to grow this material. One of the most promising methods is the use of CVD to grow graphene onto various metallic substrates. In CVD growth, graphene is grown on the surface of the metal after hydrocarbons decompose^2^. The most promising metal substrate used until now is Cu^3^, however graphene is currently grown also on Ni(111)^4–6^, Ru(0001)^7^, Pt(111)^8,9^, and Ir(111)^9–11^. Subsequently to the growth process, for graphene to be use in electronic applications, it needs to be transferred on an insulating substrate (i.e. Si/SiO_2_, quartz or polyethylene terephthalate (PET), which is a transparent and flexible substrate). The already happening now use of graphene in RF electronics^12^, integrated circuits^13^ and optoelectronics^14^ has triggered impressive progress in large-scale production, however the community still lacks standardised electrical measurements to extract useful parameters such as carrier concentration, mobility and sheet resistance, all of those are often presented as figures-of-merit of the graphene quality. Currently, the most widely used method for electrical characterisation involves magneto-transport measurements in lithographically patterned Hall bars to extract carrier concentration, mobility and resistance. However, this method is not suitable for high throughput characterisation and often the measured graphene is significantly altered due to the microfabrication processes, in which case the electrical properties of the pristine transferred graphene are different from the ones measured. An alternative method for mapping the sheet resistance of graphene films is using micro four-point probes (M4PP)^15,16^. Recently the use of microwave cavities^16–18^ and Terahertz time-domain Spectroscopy (THz-TDS)^16,19–23^ measurements provided a non-contact method of measuring the conductivity of graphene films. Despite the attractiveness of these techniques, fluctuations in ambient conditions (such as humidity) affect the electrical properties of graphene^24,25^, resulting in discriminations between measurements at different laboratories.

In contrast to the conventional Hall effect, where device fabrication is required for carrier concentration and mobility measurements, the van der Pauw (vdP) method only requires thin uniform samples with relatively small Ohmic contacts^26^. This method was originally introduced by van der Pauw in 1958 to measure the resistivity and Hall coefficient of arbitrary shape semiconductors^27,28^.

THz-TDS has recently been demonstrated to be an excellent tool to characterize the quality and conductivity of graphene deposited on large areas with suitable choices of substrates and models. THz-TDS can provide a map of the absolute value of conductivity/resistivity in a non-contact and non-destructive manner. The measurement is fast and can cover the entire area of a sample and can be used in all stages of a material development since the prototyping up to manufacturing scale^29–31^.

An important property of graphene is its work function $({\varPhi }_{Gr})$. Currently, photoemission electron microscopy (PEEM)^32^ is commonly used for measuring the local work function and distribution of graphene layers with nanometre resolution. Nevertheless, this technique requires ultra-high vacuum to operate and the surface must be completely clean, meaning that high temperature annealing (~400 °C) is used prior the measurement. Both conditions pose a significant drawback when rapid characterisation is needed. An alternative, complementary technique that can provide valuable information about local electrical properties, such as surface potential, doping and work function as well as providing essential information about layer distribution down to ~20 nm spatial resolution is frequency-modulated Kelvin probe force microscopy (FM-KPFM)^33,34^. KPFM has already demonstrated great potential for graphene characterisation, identification of graphene layers as well as measuring the surface potential of the samples with nanometre resolution^24,35–40^.

In this paper, we propose and demonstrate a standardised procedure for measuring and mapping the electrical properties of scalable graphene using a combination of contact and contactless complementary methods, such as van der Pauw magneto-transport, THz-TDS conductivity mapping and Kelvin probe force microscopy. The methodologies here described are currently being developed within the GRACE project^41^. They will become part of Good Practice Guides for the measurement of electrical properties of graphene and will be proposed as an input to normative bodies, towards the development of documentary standards. In this work, we demonstrate appropriate measurement techniques to characterise the most important electrical properties of graphene from macroscale down to nanoscale and establish the validity of the measurements through correlation in an inter-laboratory study and applying a weighting correction function between the results obtained from THz-TDS and van der Pauw methods.

## Measurements of Electrical Properties on Different Scales

### Confocal laser scanning microscopy

Confocal laser scanning microscopy (CLSM) is a powerful optical technique that enables rapid structural characterisation of large-area graphene and graphene nanostructures by pushing the spatial resolution beyond the optical diffraction limit and stacking individual images of different focal planes. CLSM was performed using an Olympus LEXT OLS4100 system equipped with 5×, 10×, 20×, 50× and 100× objectives (numerical apertures: 0.15, 0.30, 0.60, 0.95 and 0.95, respectively) and with further 1× to 8× optical zoom. Therefore, images of a field-of-view ranging from 2560 μm to 16 μm can be captured. The system uses a 405 nm wavelength laser, which scans in X-Y directions using an electromagnetic micro-electromechanical system (MEMS) scanner and a high-precision galvano-mirror. The optical images (of up to 4096 × 4096 pixels with horizontal spatial resolution down to 120 nm) are generated using a photomultiplier which captures the reflected light. In this confocal setup, in-focus reflected light is only allowed to pass through the circular confocal pinhole, therefore eliminating flare from out-of-focus areas. Due to the shallow depth-of-field of the CLSM setup, the objective is moved vertically to capture images of different focal planes. Using this Z-stacking technique, a single output of the 2D reflected intensity map is extracted by determining the maximum brightness value of the calculated *Intensity-Z* curves for each pixel from the stack of images. The system is operated in ambient air and does not require any sample preparation. For these measurements, individual images with high magnification (100 × objective) were captured and then stitched together to generate a high resolution image of a larger area^42^.

### Kelvin probe force microscopy

**Figure 1:**
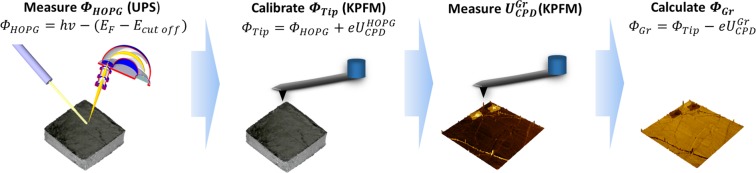
Measurement and calibration procedure for definition of local work function in graphene samples. The dark rectangles in the upmost right image (work function map) correspond to bilayer graphene with lowest values of work function.

FM-KPFM uses the force gradient $(\frac{dF}{dz})$ to calculate surface potential (${U}_{CPD}$). This is done by measuring a mechanical resonant frequency shift of a cantilever either in single or double pass. In single-pass FM-KPFM, the cantilever is oscillating at its mechanical resonant frequency *f*_0_ ≈ 300 kHz, with an AC voltage of much lower frequency *f*_*mod*_ ≈ 3 kHz of 5 V amplitude also applied, to induce a frequency shift of *f*_*0*_ ± *f*_*mod*_. The side lobes (monitored by a PID feedback loop) generated by this shift are minimized by applying a DC compensation voltage. By measuring this DC voltage at each pixel, a surface potential map [*i.e*. contact potential difference (${U}_{CPD}$)] is constructed^33,35,36^. The measurements were performed in ambient conditions (32% RH, 22 °C). Since FM-KPFM measures the surface potential (difference in work function between the tip and the sample), it provides a qualitative result, highly dependent on the tip status. In our approach we demonstrate a method to appropriately calibrate the work function of the scanning tip (${\varPhi }_{Tip})$ to quantitative measure the absolute work function of the graphene samples. The calibration method is presented in Fig. 1. Firstly, freshly cleaved highly oriented pyrolytic graphite (HOPG) is measured using ultra-violet photoelectron spectroscopy (UPS). Therefore the work function of HOPG is calculated using ${\varPhi }_{HOPG}=hv-x=4.48\,{\rm{eV}}$, where $hv=21.22\,{\rm{eV}}$ is the photon energy and $x$ is the energy difference between the Fermi edge and cut-off (for more experimental details on UPS see ref. ^24^). Then the same HOPG reference sample is measured using FM-KPFM, and the tip is calibrated using ${\varPhi }_{Tip}={\varPhi }_{HOPG}+e{U}_{CPD}^{HOPG}$, where ${U}_{CPD}^{HOPG}$ is the surface potential measured using FM-KPFM. Subsequently to the calibration, the graphene sample is measured, and the work function can be calculated using ${\varPhi }_{Gr}={\varPhi }_{Tip}-e{U}_{CPD}^{Gr}$. A good practice is to check the calibration of the tip after any measurements to ensure no drift or error.

### THz-TDS sheet resistance mapping

The extraction of the resistivity of a thin film on a substrate using THz-TDS in reflection geometry (*R*_*s*_^*THz*^) is based on measuring the difference of the THz reflectivity of the film on top of the substrate and the reflectivity of the bare substrate. In transmission geometry, the conductivity is extracted by comparing the signal of the sample with the thin film and the signal of the bare substrate in transmission mode.

The reflectivity is governed by the difference in refraction index of the film and substrate and the Fresnel equations. The relative amplitude of these two THz time-domain reflectivities analysed in the frequency-domain via a Fourier transform of each individual pulse allows estimating the contribution in the resistivity of the thin film on top of the substrate.

The THz-TDS measurement where carried out with das-Nano Onyx 2D materials quality-inspector system. This system generates maps of sheet resistance of the entire area of a sample in a non-destructive and non-contact way. The measurements are realized in normal reflection. The maps were acquired on an area of 10 × 10 mm with a step size of 100 µm and the acquisition took about 5 minutes. The maps were generated at 0.5 THz. The measurements were conducted at room temperature between 24–26 °C and relative humidity between 35–50%.

### Magneto-transport measurements in the van der Pauw geometry

**Figure 2:**
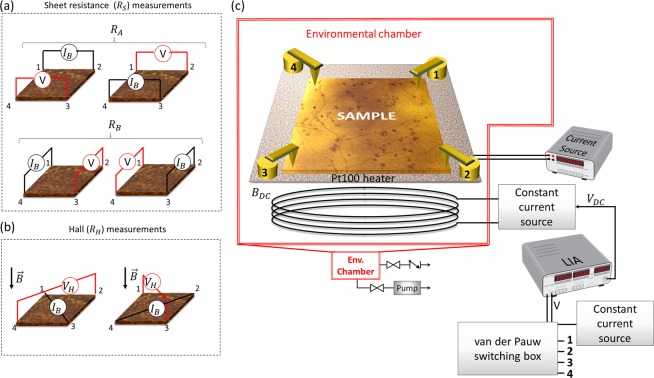
Schematics of van der Pauw configurations for (**a**) sheet resistance and (**b**) Hall effect measurements. (**c**) Experimental setup of magneto-transport measurements of un-patterned samples in the vdP geometry using AC bias and DC magnetic field. In all the cases, the sample in the schematics is represented by an experimental KPFM map of a CVD graphene sample.

VdP measurements are performed by placing contacts at the edges (periphery) of the sample, passing current and measuring voltages in different configurations^27,28^. The samples used in this work have square shape, therefore contacts are placed at the corners (see Fig. 2). The vdP method can be described by two modes of operation: (i) sheet resistance (${R}_{s}^{vdP}$) and (ii) Hall measurements (${R}_{H}$).

#### Sheet resistance measurements

By applying current and measuring the voltage drop at the opposite sides and repeating the operation by rotating the sample by 90° (Fig. 2a), 8 resistance values can be calculated:

1 $$\begin{array}{cc}{R}_{43,12}=\frac{{V}_{43}}{{I}_{12}}\, & {R}_{34,21}=\frac{{V}_{34}}{{I}_{21}}\\ {R}_{32,41}=\frac{{V}_{32}}{{I}_{41}} & {R}_{23,14}=\frac{{V}_{23}}{{I}_{14}}\\ {R}_{12,43}=\frac{{V}_{12}}{{I}_{43}} & {R}_{21,34}=\frac{{V}_{21}}{{I}_{34}}\\ {R}_{41,32}=\frac{{V}_{41}}{{I}_{32}} & {R}_{14,23}=\frac{{V}_{14}}{{I}_{23}}\end{array}\}$$

Using the values from Eq. (1), the resistances ${R}_{A}$ (current ${I}_{14}$, ${I}_{32}$) and ${R}_{B}$ (current ${I}_{21}$, ${I}_{43}$) can be derived:

2 $${R}_{A}=\frac{{R}_{43,12}+{R}_{34,21}+{R}_{12,43}+{R}_{21,34}}{4}$$

and

3 $${R}_{B}=\frac{{R}_{32,41}+{R}_{23,14}+{R}_{41,32}+{R}_{14,23}}{4}$$

Therefore, the sheet resistance ${R}_{s}^{\,vdP}$ of the graphene sample is calculated numerically using:

4 $$exp(\frac{-\pi {R}_{A}}{{R}_{s}^{vdP}})+exp(\frac{-\pi {R}_{B}}{{R}_{s}^{vdP}})=1$$

#### Hall coefficient measurements

For the Hall coefficient measurements, the sample is placed in a perpendicular magnetic field ($B$). A bias current (${I}_{B}$) is applied in two diagonals and the Hall voltage (*V*_*H*_) is measured between the remaining two corners (see Fig. 2b). To eliminate any offset voltage and magnetoresistance effects, it is important to change the direction of the current and magnetic field. The measurements for the Hall voltage are done in the following configurations, with south and north poles indicated by *S* and *N*, respectively:

5 $$\begin{array}{c}{V}_{H}^{24}:\,{I}_{13},\,{{V}_{24}}^{(S)}\,{I}_{13},\,{{V}_{24}}^{(N)}\,{I}_{31},\,{{V}_{42}}^{(S)}\,{I}_{31},\,{{V}_{42}}^{(N)}\\ {V}_{H}^{13}:\,{I}_{24},\,{{V}_{13}}^{(S)}\,{I}_{24},\,{{V}_{13}}^{(N)}\,{I}_{42},\,{{V}_{31}}^{(S)}\,{I}_{42},\,{{V}_{31}}^{(N)}\end{array}\}$$

This results in the following Hall voltage:

6 $${V}_{H}=\frac{({V}_{24}^{S}-{V}_{24}^{N})+({V}_{42}^{S}-{V}_{42}^{N})+({V}_{13}^{S}-{V}_{13}^{N})+({V}_{31}^{S}-{V}_{31}^{N})}{8}$$

Finally, the carrier concentration (*n*) and mobility (*μ*) can be calculated using the following equations from the Hall coefficient *R*_*H*_:

7 $${R}_{H}=\frac{{V}_{H}}{{I}_{B}B}=\frac{1}{en}$$

and

8 $$\mu =\frac{{R}_{H}}{{R}_{s}}$$

#### Van der Pauw instrumentation

The house-built measurement setup is capable of performing magneto-transport measurements in both ambient and vacuum, therefore providing a tool for both quick and more in-depth measurements. Figure 2c shows the experimental setup: the sample is placed on a ceramic stage with a Pt100 heater attached underneath and electrical connections are made on top, through gold-plated contacts, featuring a half sphere contact and adjustable springs. It is important to ensure that the contacts are positioned right at the edges of the sample, as this can lead to errors in the measurement^43^.

To measure the magneto-transport properties in vdP geometry, an Arduino microcontroller is used to trigger solid state relays, allowing current to pass and voltage measurements to be done at the different configurations as shown in Table 1. Initially, the sheet resistance (${R}_{s}^{vdP}$) is calculated without magnetic field by applying an AC bias (${I}_{B}=100\,{\rm{\mu }}A$, ${f}_{current}\approx 96\,\text{Hz}$) and measuring the voltage drop across the different configurations. The Hall effect is induced by an electromagnet coil that produced a DC magnetic field (15 mT). The resulting Hall voltage (*V*_*H*_) response of the AC biased sample is measured. In both ${R}_{S}$ and *V*_*H*_ measurements, the voltages are measured using a lock-in amplifier (LIA) referenced to the first harmonic of ${f}_{current}$, a technique which offers two advantages over DC: 1) eliminates the need for additional configurations and therefore reduces complexity; 2) allows minimum electrical noise in the system. The carrier concentration and mobility of the sample are calculated by using Eqs. (7) and (8).

**Table 1:** Electrical combinations for measuring sheet resistance and Hall voltage in the van der Pauw geometry in AC bias-DC magnetic field configuration.

${{\boldsymbol{R}}}_{{\bf{S}}}$ without field					${{\boldsymbol{V}}}_{{\boldsymbol{H}}}$ with field	
	${{\boldsymbol{R}}}_{{\boldsymbol{A}}}^{1,2}$	${{\boldsymbol{R}}}_{{\boldsymbol{A}}}^{3,4}$	${{\boldsymbol{R}}}_{{\boldsymbol{B}}}^{2,3}$	${{\boldsymbol{R}}}_{{\boldsymbol{B}}}^{4,1}$	${{\boldsymbol{V}}}_{{\boldsymbol{H}}}^{2,4}$	${{\boldsymbol{V}}}_{{\boldsymbol{H}}}^{1,3}$
${{\boldsymbol{I}}}_{{\boldsymbol{AC}}}^{+}$	4	2	1	3	1	4
${{\boldsymbol{I}}}_{{\boldsymbol{AC}}}^{-}$	3	1	4	2	3	2
${{\boldsymbol{V}}}_{{\boldsymbol{AC}}}^{+}$	1	3	2	4	2	1
${{\boldsymbol{V}}}_{{\boldsymbol{AC}}}^{-}$	2	4	3	1	4	3

For each resistance and Hall voltage measurement, the positive and negative terminals for the current bias and the voltage measurement are connected to the 4 contacts at the corners of the sample according to this configuration (Fig. 2a,b).

### THz-TDS map weighting for comparison with vdP measurements

The sheet resistance values ${R}_{s}^{THz}$ obtained with TDS (see Section 2.3) and ${R}_{s}^{vdP}$ obtained with the vdP method (see Section 2.4.1) can be compared to establish a correlation between these two different but complementary methods. For a given sample, the vdP measured value ${R}_{s}^{vdP}$ is a weighted average,

9 $${R}_{s}^{vdP}=\frac{1}{A}{\int }_{A}w(P)\rho (P){\rm{d}}A$$

where *ρ*(*P*) is the local sheet resistivity at point *P*, ${\rm{d}}A$ is the surface element, and ${w}_{}(P)$ is the normalized sensitivity function of the vdP method. Much work has been devoted by Koon and others^44–47^ to identify the general form of $w(P),\,\,$which depends on the (strongly non-uniform) local current distribution associated with the vdP measurement, and to the *ρ*(P) itself. The zero-order approximation for $w(P)\,\,$is ${w}_{0}(P)$, the sensitivity function for a sample of uniform resistivity. The ${w}_{0}(P)$ was computed by numerical methods, for the case of a square sample with electrodes on its corners. For this case $\,{w}_{0}(P)$ has a bell shape (see Fig. 2a in ref. ^47^), with a value of about 3.4 in the centre and vanishing on the square edges. Therefore, the resistivity near the centre affects ${R}_{s}^{vdP}$ more than that close to the sample border.

${R}_{s}^{THz}(P)$ has a constant sensitivity over $P$ being measured with a scanning technique, and as a result, the average ${R}_{s}^{THz}$ is a non-weighted quantity. However, to perform a more accurate comparison we can also compute the weighted average ${R}_{s,w}^{THz}$ of the TDS map values using the sensitivity function of the vdP measurement:

10 $${R}_{s,w}^{THz}\,=\frac{1}{A}{\int }_{A}{w}_{0}(P){R}_{s}^{THz}(P){\rm{d}}A$$

## Results and Discussion

In the following, we present and discuss results obtained on a batch of 6 samples of CVD graphene transferred on a single quartz substrate and diced into chips.

### Optical inspection

**Figure 3:**
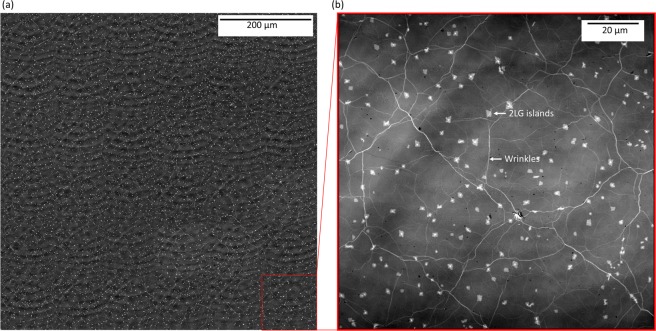
(**a**) Typical confocal laser scanning microscope optical image (pseudo colour) of a typical graphene sample (the fringe-like structures are artefacts of the method due to lens imperfections). (**b**) Zoom in area indicating 2–3LG islands and graphene wrinkles.

Figure 3 shows a confocal laser scanning microscopy image of a 700 × 700 μm area within the centre of the sample. Representative images were taken from all the samples and for different areas to ensure the continued coverage of graphene. The zoom-in image of Fig. 3b reveals details of the sample structure. It is apparent that the samples include 2–3LG islands (brighter spots). Moreover, the samples have several wrinkles due to the growth and transfer process (i.e. different thermal expansion coefficients of Cu and graphene). However, the continuity of the sample is maintained, and these features are not expected to affect the vdP or THz-TDS measurements.

### Local work function measurements

**Figure 4:**
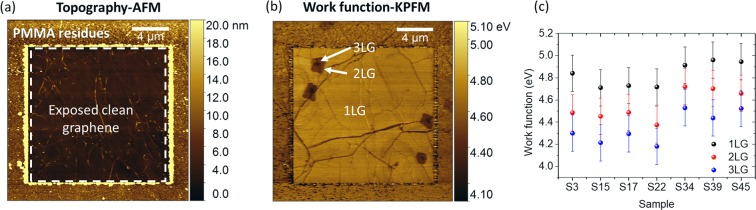
(**a**) Topography and (**b**) work function of a representative graphene sample on quartz substrate. (**c**) Summary of work functions for the different graphene samples (black, red, blue dots for 1LG, 2LG and 3LG, respectively). The PMMA residues following contact mode cleaning were accumulated in a square around the cleaned area (indicated with dashed line in (**a**)).

When a single-atomic layer such as graphene is measured, surface contamination may lead to incorrect results. When graphene is transferred on a target substrate, the top surface is covered with polymer residues. In our measurements, the residues are removed prior to the measurement using contact-mode atomic force microscopy (AFM) to expose a clean surface. The results in ambient are shown in Fig. 4a,b, where the exposed cleaned area is highlighted with white dashed lines. Following calibration of the tip (Fig. 1), the work function map was constructed (Fig. 4b), demonstrating a work function difference between the dirty and cleaned area of ~100 meV. This demonstrates the importance of cleaning the surface prior any measurements. Moreover, 2–3LG islands demonstrate lower work functions than 1LG, due to screening of the substrate charges^24,25^. The summarized results of work function measurements of the different samples are shown in Fig. 4c, with the average work functions for 1LG, 2LG and 3LG in ambient conditions being ${\varPhi }_{1LG}=4.83\,\text{eV}$, ${\varPhi }_{2LG}=4.55\,\text{eV}$ and ${\varPhi }_{3LG}=4.35\,{\rm{eV}}$, respectively.

### THz-TDS measurements

**Figure 5:**
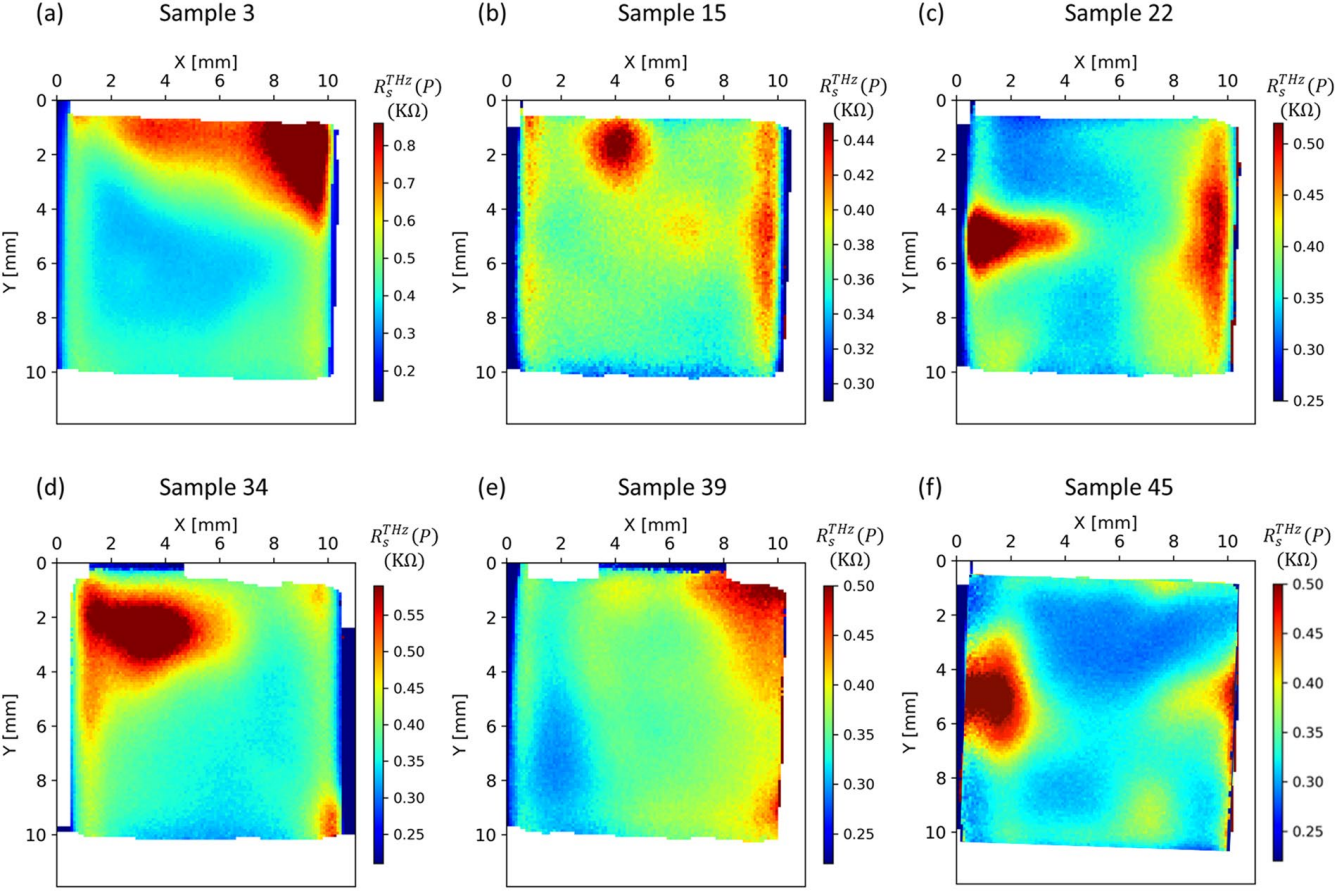
Sheet resistance maps of 6 samples obtained using the THz-TDS system, exposing areas of inhomogeneous sheet resistance.

Figure 5 shows the maps of the local sheet resistance ${R}_{s}^{THz}(P)$ obtained using the Onyx THz-TDS system. From the maps it is evident that the sheet resistance features some spatial inhomogeneities, which highlights the need for a weighted average in order to compare the values to the vdP method. Average conductance and resistance values extracted from the THz maps and their standard deviations are summarised in Table 2.

**Table 2:** Average and standard deviation of the conductance/resistance measured from THz maps. Variation coefficient is the ratio between the standard deviation and the average.

Sample	Average (mS)/(kΩ/sq)	Standard deviation (mS)/(kΩ/sq)	Variation coefficient (%)
S3	2.16/0.46	0.52/0.110	24
S15	2.64/0.38	0.13/0.019	5
S22	2.65/0.37	0.30/0.041	11
S34	2.43/0.41	0.37/0.062	15
S39	2.82/0.35	0.23/0.028	8
S45	2.69/0.37	0.33/0.052	14

The features observed in these maps have been reported in the literature in^16,48^, and they can be related to the presence of microscopic defects, such as cracks, and to the presence of growth domain. The measurements are affected by edge effects that span about 1 mm from the edge due to the size of the THz beam, which is estimated to be 0.5 mm in diameter. Most of the features show up as spots or islands of significantly higher sheet resistance, indicating a low quality of the graphene deposited in that particular area (e.g. samples 15, 22, 34 and 45). Other features show up as two distinct sheet resistance areas separated by a varying size transition zone (e.g. samples 3 and 39).

### Van der Pauw measurements

Following the THz-TDS sheet resistance mapping, the magneto-transport properties of six samples were measured using the home-build vdP setup described in Section 2.4. VdP can be considered as a complementary method to THz-TDS measurements as the later one only provided information about the sheet resistance/conductance and can only be performed in ambient conditions. As both carrier concentration and mobility are essential parameters for the material performance, their measurements provide crucial information about the quality of the graphene layer^25^. Additional advantage of the vdP method is that using the current setup both ambient and vacuum measurements can be performed and thus the effect of the environmental exposure on the electrical properties of graphene can be studied.

**Figure 6:**
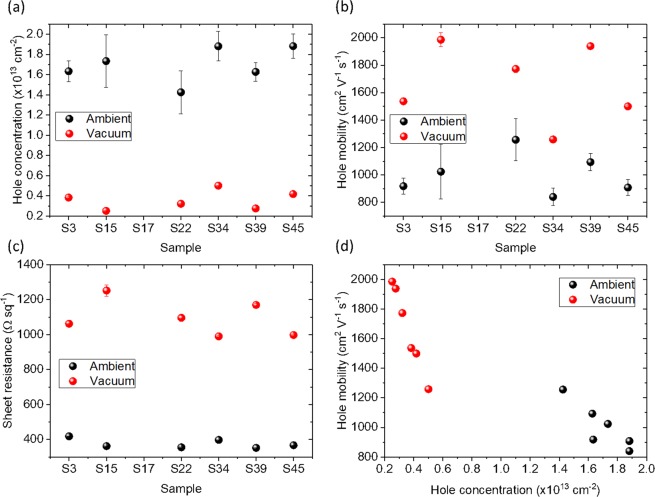
Magneto-transport measurements of the graphene samples in the van der Pauw geometry in ambient (black) and vacuum (red): (**a**) carrier concentration, (**b**) mobility, (**c**) sheet resistance for individual samples, (**d**) carrier concentration vs mobility dependence.

The results of vdP measurements are shown in Fig. 6. Initially the samples were stored in a desiccator and then they were measured in ambient (relative humidity and temperature of ~33% and ~25 °C, respectively). The samples show a p-type behaviour with an average carrier concentration of ${n}_{h}\approx 1.7\times {10}^{13}{{\rm{cm}}}^{-2}$ in ambient (Fig. 6a), with the error bar representing variations from individual measurements because of the time the samples needed to stabilize their properties. The average carrier mobility and sheet resistance are ${\mu }_{h}\approx 1000\,{{\rm{cm}}}^{2}{{\rm{V}}}^{-1}{{\rm{s}}}^{-1}$ and ${R}_{s}^{vdP}\approx 400\,{\Omega \text{sq}}^{-1}$, respectively. The carrier concentration vs mobility measurements show linear dependence with a slope of $-7.65\times {10}^{-11}$ ${{\rm{cm}}}^{4}{{\rm{V}}}^{-1}{{\rm{s}}}^{-1}$ (Fig. 6d). Although these measurements are fast and ideal for quick electrical characterisation of graphene, environmental fluctuations in the ambient air will affect the measurement, resulting in large variations in the measured values^25^. Therefore, using the dedicated measurement setup, the magneto-transport measurements in the van der Pauw geometry were also performed in vacuum. Prior the measurement, the samples were annealed (170 °C) in vacuum ($P=0.005\,{\rm{Pa}})$ for 3 hours and then they were cool down to room temperature. This resulted in desorption of atmospheric contaminants responsible for the high doping observed in ambient conditions from the graphene surface. This is evident from the significant reduction (increase) of the carrier concentration (mobility) (Fig. 6a,b), such as in vacuum the average carrier concentration and mobility were ${n}_{h}\approx 3\times {10}^{12}{{\rm{cm}}}^{-2}$ and ${\mu }_{h}\approx 1800\,{{\rm{cm}}}^{2}{{\rm{V}}}^{-1}{{\rm{s}}}^{-1}$, respectively. Importantly, the large experimental error observed in ambient was significantly reduced in vacuum, allowing stable measurements. In vacuum, the sheet resistance of the samples increased to ${R}_{s}^{vdP}\approx 1300\,{\Omega \text{sq}}^{-1}$, due to the decreased number of carriers (Fig. 6c).

### Comparison between vdP and THz-TDS characterisation

**Figure 7:**
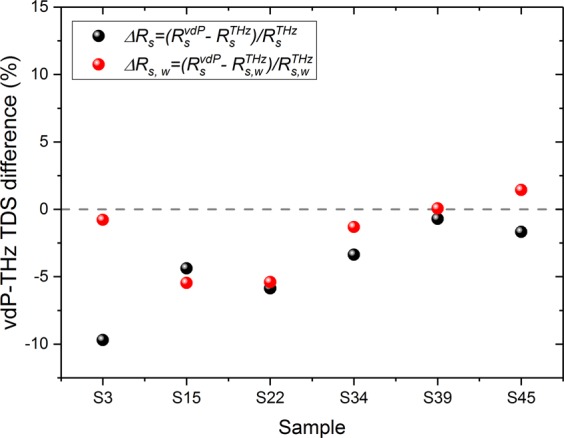
Comparison between the van der Pauw and THz-TDS methods. Black points represent the as measured difference ${\boldsymbol{\Delta }}{{\boldsymbol{R}}}_{{\boldsymbol{s}}}$, while red points represent the weighted difference ${\boldsymbol{\Delta }}{{\boldsymbol{R}}}_{{\boldsymbol{s}},{\boldsymbol{w}}}$.

In order to compare the results of vdP and THz-TDS methods, the following equations were used to calculate the relative percent differences on resistance:

12 $$\varDelta {R}_{s}=({R}_{s}^{vdP}-{R}_{s}^{THz})/{R}_{s}^{THz}$$

13 $$\varDelta {R}_{s,w}=({R}_{s}^{vdP}-{R}_{s,w}^{THz})/{R}_{s,w}^{THz}$$

where the quantities are defined by Eqs. 9-10. Although the series $\varDelta {R}_{s}$ (black points in Fig. 7 following Eq. (12)) already demonstrates a good agreement between individual values, the series $\varDelta {R}_{s,w}$ (red points following Eq. (13)) obtained after application of the vdP weighting function (Eqs. (9) and (10)) demonstrates that the residual difference between the two measurement methods is further reduced for the majority of the studied samples. For most of the samples, the relative differences are below 5%, showing a very good quantitative agreement between vdP and THz-TDS methods.

A visual explanation of the correction can be given for sample S3 as an example in Fig. 5 in which the conductivity is lower in the centre and higher around the border. Since the vdP measurement is more sensitive to the conductivity in the centre of the sample (see Section. 2.5), ${R}_{s}^{vdP} < {R}_{s}^{THz}$. Therefore, in Fig. 7, $\varDelta {R}_{s}^{\,} < 0$, and after applying the weighting corresponding to the vdP sensitivity function, the differences of sheet resistance measured between the two methods have been reduced.

## Conclusion

The characteristics of each technique are summarised in Table 3, where it is evident that a variety of complimentary techniques is necessary to obtain a complete understanding of an unknown graphene sample. While the vdP method can provide an average value of the magneto-transport properties of the graphene sample (on all insulating substrates) in both ambient and vacuum conditions in seconds, it is a contact method and discontinuities can affect the measurement significantly. On the contrary, THz-TDS mapping is a contactless method, which provides information of the spatial variations of the sheet resistance of the graphene sample, but currently it does not support measurements of carrier concentration or mobility (without the need of modeling). Also, due to the scanning procedure, it inherently takes long time (several minutes) to complete a measurement. KPFM is currently the only available method, which can provide nanometer scale work function maps of graphene. For all the methods, care must be taken when a thin film of polymer residues covers the sample. Where appropriate, it should be taken into account that values of the electrical parameters corresponding to the intrinsic properties of graphene (as measured in vacuum) can vary significantly to those measured in ambient conditions due to the presence of airborne adsorbates.

**Table 3:** Main characteristics of vdP, THz-TDS and KPFM measurement techniques.

Characteristics	Techniques		
	Van der Pauw	THz-TDS	KPFM
Type	Contact	Contactless	Contactless
Measured Quantity	Carrier concentration, mobility, sheet resistance/conductance	Sheet resistance/conductance	Work function
Environmental Conditions	Ambient, vacuum, custom	Ambient, custom	Ambient, custom, vacuum
Measurement Time	~10 seconds, though longer time for conditioning of samples in vacuum is needed	~ 1 minute	~30 minutes, depending on scan size and speed
Manufacturing Integration	Off-line batch characterisation	Wafer-scale	Off-line sample characterisation
Measurement Scale ---------------------- Spatial resolution	Global (macro) ----------------- N/A	Local (micro) ---------------------- 1 mm to 50 µm	Local (nano) --------------------- 10 to 30 nm
Sources of error	Non-ohmic contacts, discontinuities, thermoelectric effects, ambient conditions stability	Roughness of the back side of the sample	Lack of grounding, contaminated sample, wrong calibration

In conclusion, we introduce a procedure for performing electrical measurements of CVD graphene from macro- to nano-scale using a variety of contact and contactless techniques. The sheet resistance, carrier concentration and mobility were measured using macroscale magneto-transport measurements in the vdP geometry, both in vacuum and ambient conditions to extract the pristine electrical properties of graphene. Upon annealing in vacuum, the carrier concentration has significantly decreased due to desorption of atmospheric molecules (such as water), while the carrier mobility and sheet resistance have significantly increased. The measurements were compared to the sheet resistance maps obtained using microscale THz-TDS mapping, where a weighted average function was applied. For most of the samples, the difference in the sheet resistance as obtained by vdP and THz-TDS was below 5%, demonstrating the good agreement between the different methods. A method for calibrated KPFM measurements of graphene samples is demonstrated, allowing high resolution mapping of the work function on the nanoscale and highlighting the impact of polymer residues on the surface. This work highlights the importance of using a standardized methodology for electrical measurements using a variety of complimentary techniques to obtain a complete understanding of the sample on a variety of lateral scales. The development of these presented metrology and methodologies within the GRACE project^41^ will become an important part to contribute to the standards development work of the normative bodies, through the initiation of and dissemination of new written standards for the electrical characterisation of graphene based on the Good Practice Guides developed within the project.

## Methods

### Graphene growth

The mono-layer graphene growth was carried out in a cold-walled chemical vapour deposition (CVD) reactor (Aixtron BM) using Cu foil as the catalyst. Following the growth, PMMA was spin-coated onto the graphene-covered Cu foil, acting as a sacrificial support layer. The Cu foil was then etched using a FeCl_3_ containing solution. Distilled water was used to clean the film several times after it was transferred onto a quartz substrate. Finally, the PMMA layer was removed by immersion in acetone. In this work we use 4″ CVD graphene sample, which was diced into 10 × 10 mm^2^ squares. Several diced chips were chosen in random and studied by a number of methods as discussed.

## Supplementary information

Supplementary Information.

## Data Availability

The datasets generated during and/or analysed during the current study are available from the corresponding author on reasonable request.
