# Supplementary material for: Towards standardisation of contact and contactless electrical measurements of CVD graphene at the macro-, micro- and nano-scale
Source: Sci Rep. 2020 Feb 21;10:3223. doi: 10.1038/s41598-020-59851-1 (PMC7035257; doi:10.1038/s41598-020-59851-1)
Supplement: Supplementary file 1 — Supplementary Information. [file 41598_2020_59851_MOESM1_ESM.docx]

**Supplementary Information:**

**Towards standardisation of contact and contactless electrical measurements of CVD graphene at the macro-, micro- and nano-scale**

Christos Melios^1^, Nathaniel Huang^1^, Luca Callegaro^2^, Alba Centeno^3^, Alessandro Cultrera^2^, Alvaro Cordon^4^, Vishal Panchal^1^, Albert Redo-Sanchez^4^, David Etayo^4^, Montserrat Fernandez^4^, Alex Lopez^4^, Sergiy Rozhko^1^, Oihana Txoperena^3^, Amaia Zurutuza^3^, and Olga Kazakova^1^

^1^National Physical Laboratory, Teddington, TW11 0LW, United Kingdom

^2^Istituto Nazionale di Ricerca Metrologica, Strada delle Cacce 91, 10135 Torino, Italy

^3^Graphenea SA, 20018 Donostia-San Sebastián, Spain

^4^Das-Nano, Poligono Industrial Talluntxe II, Calle M-10, 31192 Tajonar, Navarra, Spain

**Frequency dependent performance of the van der Pauw system**

For AC measurements, it is important to understand the frequency dependence of the measurement setup. AC measurements using LIA’s are considerably less noisy compared to DC. However, care must be taken when choosing the biasing frequency, as low frequencies might induce larger noise (i.e. 50 Hz from mains, including harmonics and 1/*f* noise), but at higher frequencies parasitic effects (such as inductance and capacitance) will significantly increase spurious voltage pickup. Therefore, an appropriate frequency needs to be chosen, where it is high enough to eliminate low frequency noise, but low enough to exclude high frequency effects.

The frequency performance of the van der Pauw system was characterised by performing frequency dependant measurements of the resistance, Hall voltage and the output parameters such as carrier concentration, mobility and sheet resistance. The measurements were performed on a CVD graphene sample transferred on a quartz substrate. The measurements were performed at 24 °C and 38% relative humidity. The samples were biased using AC of 50 μA (peak amplitude) and the Hall measurements were performed at 15 mT DC magnetic field. The LIA time constant was kept at 300 ms, input was A-B mode, AC coupling, normal reserve and 18 dB slope/octave.


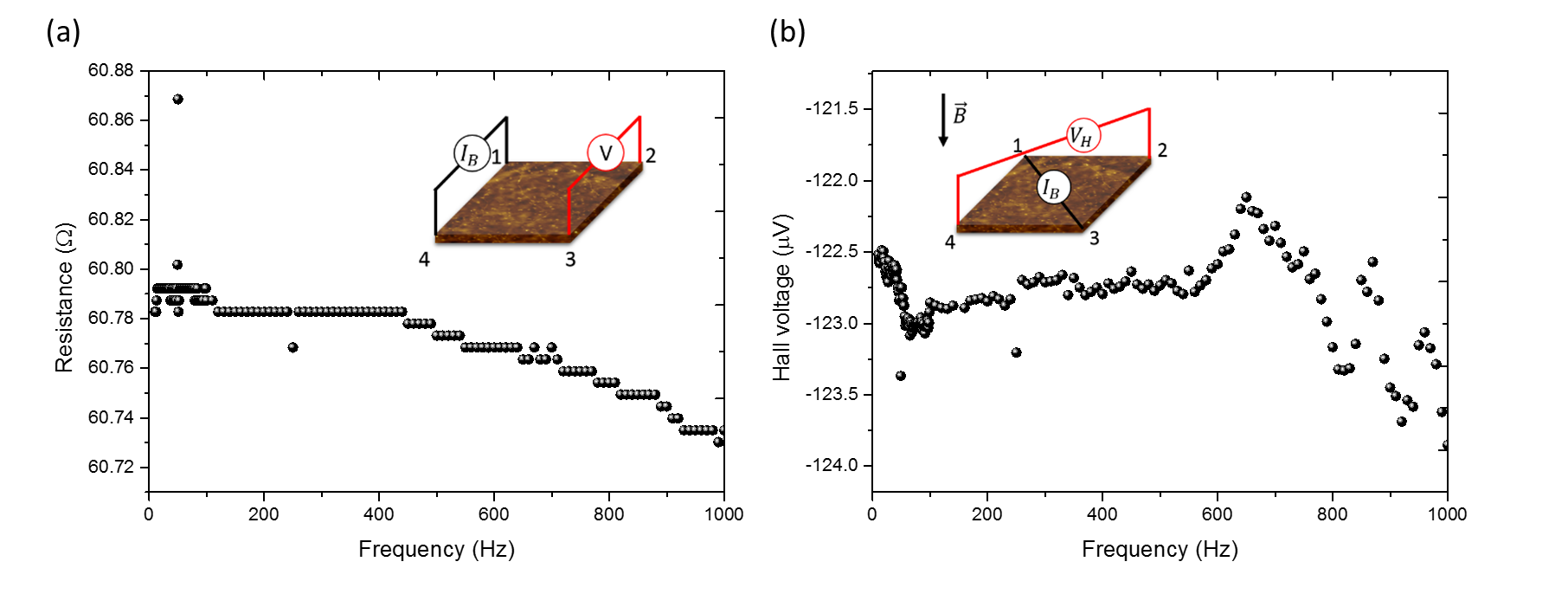


Figure S1: Frequency dependence of the (a) resistance and (b) Hall voltage in the two geometries shown in the insets.

Figure S1 shows the frequency dependence of resistance and Hall voltage changes. Below 100 Hz, the resistance appears to be noisier compared to mid-frequency range (100-450 Hz). However, in the mid-frequency range, particularly at 400 Hz, the resistance appears to be frequency independent. At frequencies above 450 Hz, the resistance exhibits a frequency dependence, which further increases with higher frequencies, therefore the Hall voltage suffers significantly from high frequency noise.

The frequency dependence of electrical parameters is more apparent at the measured quantities of carrier concentration, mobility and sheet resistance (Figure S2). In all measurements, the system demonstrates consistent performance up to 500 Hz. At higher frequencies, the measured values start to deviate significantly from the true values. This is more apparent on the standard deviations of the measurements, which increase dramatically above 500 Hz (see Figure S2b and d). It is therefore recommended that the frequency dependence of unknown samples is investigated prior any measurements are done.


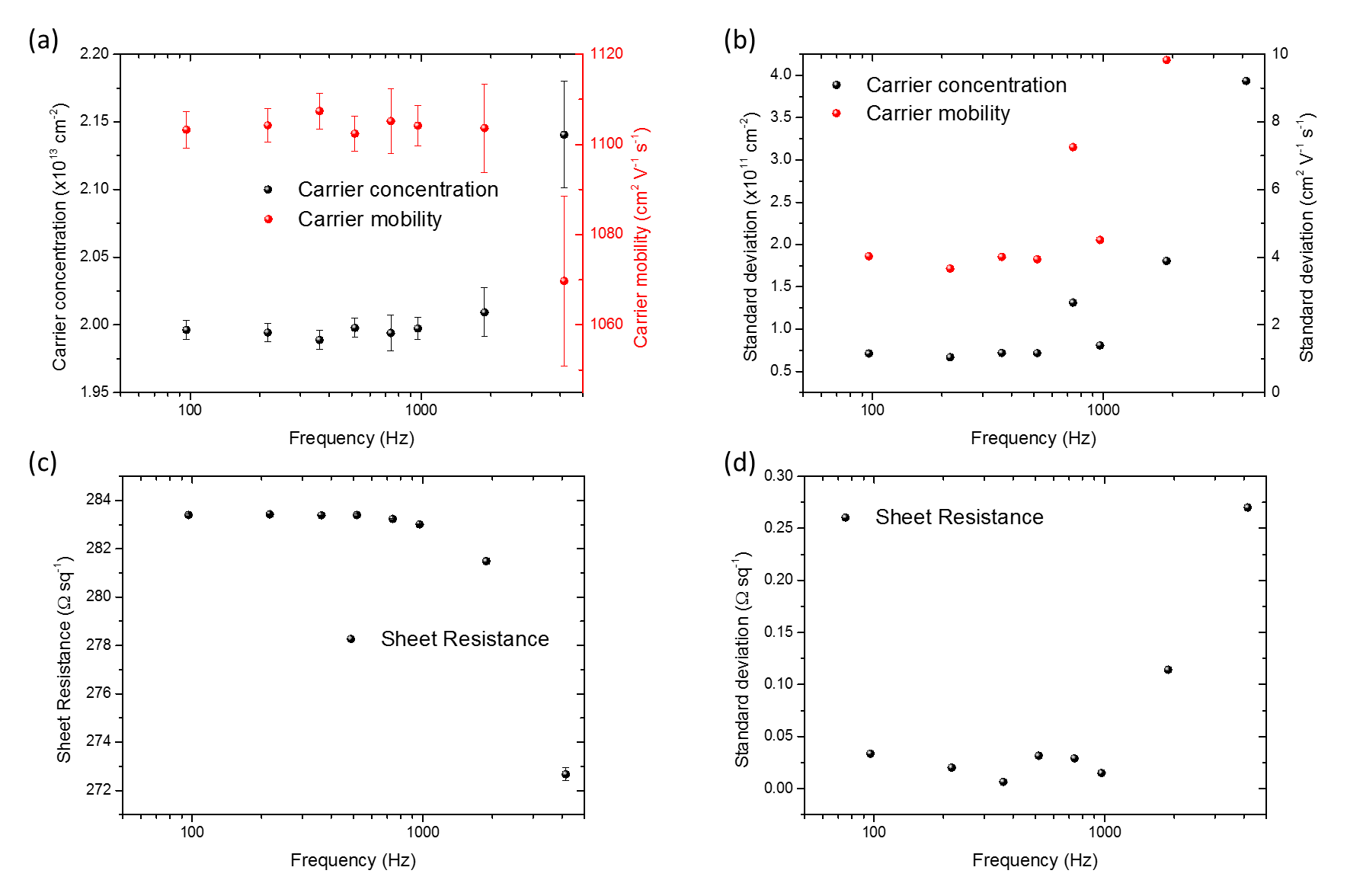


Figure S2: (a) Frequency dependence of carrier concentration (black) and mobility (red) and their respective (b) standard deviations. (c) Frequency dependence of sheet resistance and its (d) standard deviation.
